# The association between multimorbidity and health-related quality of life: a cross-sectional survey among community middle-aged and elderly residents in southern China

**DOI:** 10.1186/s12955-019-1175-0

**Published:** 2019-06-24

**Authors:** Xin-Yu Bao, Yi-Xian Xie, Xiao-Xia Zhang, Xin Peng, Jun-Xuan Huang, Qing-Feng Du, Pei-Xi Wang

**Affiliations:** 1 Department of Preventive Medicine, School of Public Health, Guangzhou Medical University, Guangzhou, Guangdong 510182 People’s Republic of China; 2 Institute of Chronic Disease Risks Assessment, School of Nursing and Health, Henan University, Kaifeng, 475004 China; 3 General Practice Center, Nanhai Hospital. Southern Medical University, Foshan, 528000 Guangdong China

**Keywords:** Health-related quality of life, Multimorbidity, Middle-aged, Elderly, EQ-5D

## Abstract

**Background:**

Multimorbidity is common among the middle-aged and elderly residents. And it is associated to the reduction of health-related quality of life (HRQoL), including physical and psychological dimensions. However, there are few studies that have paid attention to the HRQoL of residents with multimorbidity in China. Therefore, this study aims to investigate the relationships between different multimorbidity patterns and HRQoL among middle-aged and elderly adults in China.

**Methods:**

Based on a cross-sectional survey, the information regarding 18,137 adults, who were at least 45 years of age, was collected through interviews. Self-perceived HRQoL was assessed with the EQ-5D-3 L instrument, and the EQ-5D-3 L index score was calculated using the Chinese EQ-5D-3 L value set. The Tobit regression was used to explore the impacts of multimorbidity groups on HRQoL.

**Results:**

Of 18,137 respondents, more than a fifth (3773,20.8%) of people had multimorbidity. Mean (SD) of EQ-5D index and VAS values were 0.95(0.14) and 76.02(13.66), respectively. Significant correlations were found between a lower HRQoL and increasing numbers of chronic conditions (*P* < 0.001). Most of chronic diseases co-occurred frequently, and the association between hypertension and diabetes mellitus was the strongest (adjusted OR = 3.82). The most prevalent disease is hypertension (5052,27.9%), and the most prevalent chronic diseases pair is hypertension and diabetes mellitus (841,4.6%). Among those chronic diseases with high prevalence, the effects on HRQoL ranged from chronic pain to hypertension (adjust b = − 0.036 to − 0.008). In the common multimorbidity patterns, co-occurrence of chronic pain and bone disease (adjust b = − 0.039) had the greatest impact on HRQoL.

**Conclusions:**

The HRQoL of middle-aged and elderly adults declines by multimorbidity. More attention should be paid to the HRQoL of residents with multimorbidity in China. The effect of different multimorbidity patterns on HRQoL is not simply added by two diseases, but changes by the different combination. Identifying different multimorbidity patterns of residents can provide more targeted measures to improve the HRQoL.

## Background

Multimorbidity, commonly defined as the co-existence of two or more chronic conditions in one person [1, 2], is related to increased rates of mortality and disability, reduced function levels, increased the burden of disease and treatment, which can affect daily activities and well-being, including loss of independence and autonomy. And these factors cause more poor HRQoL of multimorbid patients than those of the general population [3, 4]. Chronic diseases are often viewed as primarily affecting old people. In fact, one quarter of death from chronic disease occur in people under 60 years of age [5]. Moreover, the prevalence of multimorbidity is widespread among working-age adults (20–64 years old) around the world [6]. The impact of multimorbidity is more serious than we imagined.

Several studies have shown that multimorbidity is significantly associated with poor HRQoL in adults [4, 7]. However, in China, a country with high prevalence of multimorbidity [8], residents with multimorbidity are not received enough attention about their HRQoL. Although some studies have shown that the HRQoL of residents declines as the number of chronic diseases increases, in China [9]. However, compared with other countries [10], there is no further study on the impacts of different multimorbidity patterns on HRQoL, which requires us to improve.

There are many tools for measuring the quality of life, such as SF-36, ADL, and GHQ. And the EQ-5D is one of the most frequently used instruments to assess HRQoL in health economic evaluation [11], perhaps the most appropriate tool for measuring the quality of life of the population across the board [12, 13]. It provides a simple descriptive profile and a single index for health status that can be used in the clinical and economic evaluation of healthcare, as well as in population health surveys [14].

Therefore, regarding the increasing importance of multimorbidity and the lack of studies regarding the relationship between multimorbidity and HRQoL, we conducted a cross-sectional study among the adults aged ≥45 years of age in Southern China. The study aims to confirm the effect of multimorbidity on HRQoL, observes the association between two chronic diseases, and explores the impacts of different multimorbidity patterns on HRQoL.

## Methods

### Data source

This study was based on a cross-sectional community health survey in the Foshan city of Guangdong province in Southern China, which have characteristics similar to the national average in terms of population demographics. The samples in this survey were selected using a multistage and stratified random sampling method. The stratification according to the level of the economy and randomly selected street communities at economic levels, then randomly selected communities in street communities according to proportion. Households within residential communities were then randomly selected from the household lists. In the present study, we used the available information from people aged ≥45 years of age. Of 19,037 participants, 900 (4.73%) subjects were excluded due to incomplete or inconsistent data from questionnaires, including reluctance to provide part of their personal information or not aware of their chronic diseases. Finally, a total of 18,137 middle-aged and elderly adults were included in our analyses.

## Measure

### The EQ-5D-3 L

It is a generic HRQoL measure which can compare HRQoL in populations [7]. The Chinese version of EQ-5D-3 L scale has good reliability and validity [15]. The EQ-5D-3 L consists of two parts: a health description system and Visual Analogue Scale (VAS). The first part records self-assessed health status according to five dimensions: mobility, self-care, usual activities, pain/discomfort, and anxiety/depression. Each dimension is divided into three levels: no problem, some problems, and extreme problems. A total of 243 health state can be expressed by combining the different level from each dimension. This is then transformed into a weighted health state index score (EQ-5D index) by the Chinese time trade-off value, which is a conversion weight from health utility measurements designed based on the HRQoL preferences of populations. And the range of EQ-5D index is − 0.149 to 1 [16]. The second part used to assess self-perceived levels of health from 0 to 100, where 0 represents the worst imaginable state of health and 100 represents the best imaginable state.

### Chronic disease measure

The presence of chronic diseases was determined by self-report, as a yes/no response to the question stem, “has a doctor ever diagnosed that you had...” [17]. The 19 diseases investigated in this study included: hypertension, chronic pain, bone diseases, diabetes mellitus, dyslipidemia, gastroenteritis, gout, coronary disease, peripheral vascular disease, stroke, spleen and gallbladder diseases, chronic obstructive pulmonary disease, chronic kidney disease, cancer, multiple sclerosis, dementia, anxiety, depression and phthisis.. In the face-to-face interview the trained interviewer was used to verify the chronic illness of the interviewees through the diagnosis results and medication to ensure the authenticity and comprehensiveness of the interview.

### Participants’ characteristics

The variables used for the analyses included age, gender, marital status, education level, and employment status. Marital status was divided into two categories: currently married and single (including unmarried, divorced or widowed). Education level was grouped into three categories as follows: primary school or lower, middle school, high school or above. Employment status was categorized into employed and unemployed. In this study, the definition of unemployed included all subjects who were without work [18].

### Statistical analysis

First, descriptive statistics were calculated for all measures. Means and standard deviations (SD) were presented for continuous variables, while frequency and percentage were used for categorical variables. Second, we combined the groups “some problems” and “extreme problems” into one group, called “problem” group, and compared it with “no problem” group. We evaluated the proportion of having problem on five dimensions and average EQ-5D index. The univariate logistic regression and univariate Tobit regression were used to examine the differences among the category of demographic variable.

Then, we displayed 19 chronic diseases by calculating the prevalence of each disease individually. We chose the prevalent chronic medical conditions with a prevalence > 4%, and analyzed their correlations using contingency tables to generate the most prevalent multimorbidity pairs (the combination of two chronic diseases). And the logistic regression was used to analyze the association between two chronic diseases in the pairs that was controlled for demographics.

Finally, we explored the impacts of multimorbidity pairs on HRQoL. The ceiling effect, which is defined as when the scales are developed such that substantial proportions of individual obtain maximum score and the true extent of their abilities cannot be determined, is particularly evident with the EQ-5D index [19, 20]. Previous researches have shown that preference-based HRQoL index scores exhibit ceiling effects, with a significant number of respondents reporting the highest score (1.0) [9]. In these circumstances the censored least absolute deviations estimator developed by Powell and Tobit models have theoretical advantages over the ordinary least squares estimator [21–23]. In our research, more than 80% people with “no problem” which means all of them got highest score (1.0) and Tobit regression was used as well as the marginal disutility estimates were reflected by the regression.

Statistical analyses were completed using SPSS version 20 software and Stata V.15.0 software. Values of *P* < 0.05 were taken to indicate statistical significance.

## Result

In our analyses, the total of 18,137 participants aged 45 years and above were included. Table 1 presents the socio-demographic characteristics of this sample. The average age was (61.36 ± 10.79) years old. Female and married people accounted for 52.4 and 88.0% of study respondents, respectively. Among them, most received an education of primary school or lower, and 37.8% of individuals were still employed. 48.3% had at least one chronic disease, and more than a fifth of the study population had multimorbidity. The prevalence of multimorbidity was 12.8% in the middle-aged (45–64 years old) and 33.8% in older adults (over 65 years old). The average number of chronic conditions was (0.80 ± 1.04). The EQ-5D VAS value was (76.02 ± 13.66), with a minimum of 0 and a maximum of 100. The EQ-5D index value was (0.95 ± 0.14). 81.6% of people reported self-perceived no problem in all five dimensions and their EQ-5D index score get 1.0.

**Table 1:** Descriptive statistics of the study sample

		Sample(*N* = 18,137)
Age, mean (SD)		61.36 (10.79)
Age group, years, n (%)	45~54	5915 (32.6)
	55~64	5286 (29.1)
	65~74	4722 (26.0)
	75~	2214 (12.2)
Gender, n (%)	Male	8637 (47.6)
	Female	9500 (52.4)
Marital status, n (%)	Single ^a^	2173 (12.0)
	Married	15,964 (88.0)
Education level, n (%)	Primary school or lower	15,784 (87.0)
	Middle school	1745 (9.6)
	High school or above	608 (3.4)
Employment status, n (%)	Unemployed	11,282 (62.2)
	Employed	6855 (37.8)
Number of chronic diseases, n (%)	0	9376 (51.7)
	1	4988 (27.5)
	2	2329 (12.8)
	3	906 (5.0)
	> = 4	538 (3.0)
EQ-5D vas, mean (SD)		76.02 (13.66)
EQ-5D problems, n (%)	Some problems	3337 (18.4)
	No problem	14,800 (81.6)
EQ-5D index, mean (SD)		0.95 (0.14)

^a.^Single: unmarried, divorced, widowed

Then the comparisons of proportion with problems in five dimensions and EQ-5D index average score are showed in Table 2. Residents had the highest proportion of some or extreme problems in the pain/discomfort dimension (13.8, 95% *CI* 13.3 to 14.3%). A decrease in EQ-5D index with age was highly significant (*P* < 0.001) in the study results. Compared with residents without chronic disease, residents with 1, 2, 3 and > =4 chronic diseases had higher proportions of some or extreme problems in the five dimensions, respectively (OR: mobility, 2.89, 5.44, 8.48 and 13.97; self-care, 2.97, 5.45, 8.09 and 12.05; activity, 3.05, 5.46, 8.84 and 14.97; pain/discomfort, 3.16, 5.90, 8.63 and 14.51; anxiety/depression, 2.33, 3.66, 6.16 and 8.93). Significantly lower values of HRQoL on EQ-5D Index was associated with female, single, lower education level, unemployed, and multimorbidity of chronic disease. And the proportion of having problems in all five dimensions significantly increased as the number of diseases increased. However, in the dimension of anxiety/depression, the proportion of having problems is highest when two chronic diseases are co-occurred.

**Table 2:** Comparisons of proportions of having problems and EQ-5D index average score

Variables	Mobility proportion % (95% CI)	Self-care proportion % (95% CI)	Activity proportion % (95% CI)	Pain/Discomfort Proportion % (95% CI)	Anxiety/Depression proportion % (95% CI)	Index score, mean (95% CI)
Total	10.7 (10.2,11.1)	7.8 (7.4,8.2)	9.0 (8.6,9.4)	13.8 (13.3,14.3)	5.1 (4.8,5.4)	0.945 (0.943,0.947)
Age group						
45~54	1.6 (1.2,1.9)	1.3 (1.0,1.6)	1.5 (1.2,1.8)	5.0 (4.4,5.5)	2.5 (2.1,2.9)	0.986 (0.984,0.987)
55~64	5.5 (4.9,6.1) *	3.4 (3.0,3.9) *	4.2 (3.7,4.8) *	10.3 (9.5,11.1) *	3.8 (3.3,4.4) *	0.968 (0.965,0.971) *
65~74	13.8 (12.8,14.8) *	9.5 (8.65,10.3) *	11.5 (10.6,12.4) *	18.4 (17.3,19.5) *	6.0 (5.3,6.6) *	0.932 (0.927,0.936) *
75~	40.7 (38.7,42.8) *	32.2 (30.2,34.1) *	34.9 (32.9,36.9) *	36.0 (34.0,38.0) *	13.2 (11.8,14.6) *	0.814 (0.804,0.823) *
Gender						
Male	9.5 (8.8,10.1)	7.0 (6.5,7.6)	8.1 (7.5,8.62)	11.8 (11.1,12.4)	4.4 (4.0,4.9)	0.952 (0.949,0.954)
Female	11.8 (11.1,12.4) *	8.5 (8.0,9.1) *	9.8 (9.2,10.4) *	15.7 (14.9,16.4) *	5.7 (5.3,6.2) *	0.940 (0.937,0.943) *
Marital status						
Single	27.0 (25.1,28.8)	20.6 (18.9,22.3)	22.7 (21.0,24.5)	28.4 (26.5,30.2)	10.6 (9.3,11.9)	0.871 (0.862,0.880)
Married	8.5 (8.0,8.9) *	6.1 (5.7,6.45) *	7.1 (6.7,7.5) *	11.8 (11.3,12.3) *	4.4 (4.0,4.7) *	0.956 (0.954,0.958) *
Education level						
Primary school or lower	11.6 (11.1,12.1)	8.5 (8.09,8.96)	9.7 (9.3,10.2)	14.9 (14.3,15.4)	5.4 (5.1,5.8)	0.941 (0.939,0.943)
Middle school	4.1 (3.1,5.0) *	3.0 (2.2,3.8) *	3.7 (2.8,4.6) *	7.2 (6.0,8.4) *	3.0 (2.2,3.8) *	0.976 (0.972,0.980) *
High school or above	4.6 (2.9,6.3) *	3.6 (2.1,5.1) *	4.1 (2.5,5.7) *	5.4 (3.6,7.2) *	3.3 (1.9,4.7) *	0.975 (0.967,0.983) *
Employment status						
Unemployed	15.7 (15.0,16.4)	11.6 (11.0,12.2)	13.3 (12.6,13.9)	18.5 (17.8,19.2)	6.7 (6.3,7.2)	0.923 (0.920,0.926)
Employed	2.4 (2.1,2.8) *	1.6 (1.3,1.9) *	1.9 (1.6,2.2) *	6.1 (5.5,6.7) *	2.4 (2.1,2.8) *	0.983 (0.981,0.985) *
Number of chronic diseases,						
0	4.4 (4.0,4.8)	3.1 (2.8,3.5)	3.5 (3.2,3.9)	5.7 (5.2,6.2)	2.5 (2.2,2.8)	0.977 (0.975,0.979)
1	11.79 (10.9,12.7) *	8.8 (8.0,9.6) *	10.0 (9.2,10.8) *	16.0 (15.0,17.0) *	5.6 (4.9,6.2) *	0.939 (0.935,0.943) *
2	20.1 (18.5,21.7) *	15.0 (13.5,16.4) *	16.6 (15.1,18.1) *	26.2 (24.4,28.0) *	28.2 (25.2,31.1) *	0.900 (0.893,0.907) *
3	28.2 (25.2,31.1) *	20.8 (18.1,23.4) *	24.4 (21.6,27.2) *	34.2 (31.1,37.3) *	13.5 (11.2,15.7) *	0.860 (0.846,0.873) *
> =4	39.2 (35.1,43.4) *	28.1 (24.3,31.9) *	35.3 (31.3,39.4) *	46.7 (42.4,50.9) *	18.4 (15.1,21.7) *	0.799 (0.779,0.819) *

*CI* Confidence interval

The first category of each demographic variable was selected as reference to do univariate logistic regression or univariate Tobit regression

*p < 0.05

Table 3 displays the rank prevalence of 19 self-reported chronic diseases and the most prevalent related pairs of these diseases with a prevalence > 4% in the sample population. The most common chronic disease in the population was hypertension (27.9%), followed by chronic pain (10.1%) and bone disease (10.0%). In addition, diabetes mellitus and dyslipidemia had a prevalence of 7.5 and 6.0%, respectively. The most prevalent chronic disease pair was hypertension and diabetes mellitus (4.6%), followed by hypertension and bone disease (3.9%), and hypertension and chronic pain (3.7%).

**Table 3:** The rank prevalence of 19 self-reported chronic diseases and most prevalent related pairs of chronic diseases with a percentage > 4% in the sample population

Type of single disease	Sample,n (%)	95% CI	Rank	Pairs of diseases^b^	Sample,n (%)	95% CI
Hypertension	5052 (27.9)	27.2,28.5	1	HT + DM	841 (4.6)	4.3,4.9
Chronic pain	1827 (10.1)	9.6,10.5	2	HT + BD	712 (3.9)	3.6,4.2
Bone disease	1821 (10.0)	9.6,10.5	3	HT + CP	663 (3.7)	3.4,3.9
Diabetes mellitus	1361 (7.5)	7.1,7.9	4	HT + DL	628 (3.5)	3.2,3.7
Dyslipidemia	1084 (6.0)	5.6,6.3	5	CP + BD	498 (2.8)	2.5,3.0
Gastroenteritis	782 (4.3)	4.0,4.6	6	HT + GT	417 (2.3)	2.1,2.5
Gout	775 (4.3)	4.0,4.6	7	DM + DL	231 (1.3)	1.1,1.4
Coronary disease	449 (2.5)	2.3,2.7	8	HT + GE	216 (1.2)	1.0,1.4
Stroke	426 (2.4)	2.1,2.6	9	CP + DL	209 (1.2)	1.0,1.3
Peripheral vascular disease	411 (2.3)	2.1,2.5	10	BD + DL	208 (1.2)	1.0,1.3
Spleen and gallbladder diseases	255 (1.4)	1.2,1.6	11	CP + GE	205 (1.1)	1.0,1.3
COPD^a^	187 (1.0)	0.9,1.2	12	BD + DM	180 (1.0)	0.9,1.1
Chronic kidney disease	125 (0.7)	0.6,0.8	13	BD + GE	165 (0.9)	0.8,1.1
Cancer	104 (0.6)	0.5,0.7	14	BD + GT	153 (0.8)	0.7,1.0
Multiple sclerosis	52 (0.3)	0.2,0.4	15	DL + GT	146 (0.8)	0.7,0.9
Dementia	45 (0.3)	0.2,0.3	16	CP + DM	144 (0.8)	0.7,0.9
Anxiety	37 (0.2)	0.1,0.3	17	CP + GT	128 (0.7)	0.5,0.8
Depression	27 (0.2)	0.1,0.2	18	DM + GT	87 (0.5)	0.4,0.6
Phthisis	22 (0.1)	0.1,0.2	19	DL + GE	79 (0.4)	0.3,0.5

*CI* Confidence interval, *HT* Hypertension, *CP* Chronic pain, *BD* Bone diseases, *DM* Diabetes mellitus, *DL* Dyslipidemia, *GE* Gastroenteritis, *GT* Gout

^a^ COPD, chronic obstructive pulmonary disease

^b^ All pairs have significantly correlation (*P* < 0.05)

**Fig. 1:**
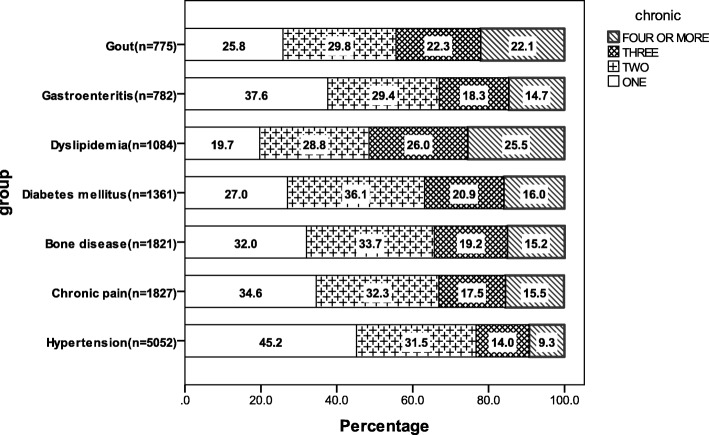
Distributions of chronic conditions

Of those who reported any chronic diseases (*n* = 8761), 27.5% (*n* = 4988) reported experiencing only one condition. Among those comparatively common chronic conditions, the proportion of experiencing one condition with an additional chronic disease was highest in multimorbidity distribution. The condition with the least reported multimorbidity was hypertension, 45.2% of these participants only experienced it alone. On the contrary, those with dyslipidemia reported experiencing more multiple long-term limited conditions, with 80.3% reporting experiencing two or more additional chronic conditions (Fig. 1).

Table 4 shows the association between two chronic diseases in the pair. Most odds ratios are statistically significantly higher than 1.0. The higher odds ratios (adjusted OR [AOR] > 3.00) were found for the chronic disease pairs of hypertension with diabetes mellitus (AOR = 3.82), hypertension with dyslipidemia (AOR = 3.80), dyslipidemia with gout (AOR = 3.80), chronic pain with bone disease (AOR = 3.64), chronic pain with gastroenteritis (AOR = 3.46), and diabetes mellitus with dyslipidemia (AOR = 3.48). The pair of diabetes mellitus with gastroenteritis (AOR = 0.66) was significantly inverse correlated.

**Table 4:** The associations between co-occuring chronic diseases

Type of disease	Chronic pain^b^		Bone disease^b^		Diabetes mellitus^b^		Dyslipidemia^b^		Gastroenteritis^b^		Gout^b^	
	OR	AOR	OR	AOR	OR	AOR	OR	AOR	OR	AOR	OR	AOR
Hypertension^a^	1.54^c^	1.28^c^	1.77^c^	1.32^c^	4.83^c^	3.82^c^	3.93^c^	3.80^c^	0.99	0.96	3.20^c^	2.76^c^
Chronic pain^a^			4.25^c^	3.64^c^	1.06	0.90	2.28^c^	2.18^c^	3.45^c^	3.46^c^	1.82^c^	1.78^c^
Bone disease^a^					1.41^c^	1.11	2.27^c^	2.12^c^	2.54^c^	2.56^c^	2.31^c^	2.21^c^
Diabetes mellitus^a^							3.82^c^	3.48^c^	0.67^c^	0.66^c^	1.60^c^	1.37^c^
Dyslipidemia^a^									1.83^c^	1.82^c^	4.06^c^	3.80^c^
Gastroenteritis^a^											1.79^c^	1.77^c^

Logistic regression models were respectively built and gender, age, education level, marriage state, and employment were adjusted in the logistic regression model

^a^ Dependent variable; ^b^ Independent variable; ^c^ significant at 0.05

Regression results reflecting marginal disutility estimates for EQ-5D index are presented in Table 5. These estimates reflected the marginal impact of experiencing the given condition, controlling for gender, age, education level, marriage, employment, and all the other chronic diseases. For example, compared with those without hypertension, the marginal impact of having hypertension resulted in a decrease of − 0.008 in the EQ-5D index, holding all other effects constant. In the same way, holding all other effects constant, the combination of hypertension and diabetes mellitus compared with those who did not have those two chronic conditions resulted in a reduction of − 0.013 in the EQ-5D index. The significant marginal impact in single chronic disease was chronic pain (− 0.036), and multimorbidity pairs were the combination of chronic pain and bone disease (− 0.039).

**Table 5:** Regression results of single chronic diseases and multimorbidity pairs on EQ-5D index

Type of disease	Crude b ^a^	Adjusted b	SE	P	95% CI
Hypertension	−0.056	−0.008	0.002	< 0.001	− 0.012, − 0.004
Chronic pain	− 0.066	− 0.036	0.003	< 0.001	− 0.042, − 0.031
Bone disease	− 0.067	− 0.030	0.003	< 0.001	− 0.035, − 0.024
Diabetes mellitus	− 0.042	− 0.010	0.003	0.002	− 0.016, − 0.003
Dyslipidemia	− 0.037	− 0.003	0.004	0.369	− 0.010, 0.004
Gastroenteritis	−0.032	− 0.014	0.004	0.001	−0.022, − 0.006
Gout	− 0.057	−0.023	0.004	< 0.001	−0.030, − 0.015
HT + DM	− 0.055	− 0.013	0.004	0.001	− 0.020, − 0.005
HT + BD	− 0.081	−0.021	0.004	< 0.001	−0.028, − 0.013
HT + CP	− 0.083	− 0.027	0.004	< 0.001	− 0.035, − 0.019
HT + DL	− 0.053	−0.006	0.004	0.157	−0.015, 0.002
CP + BD	−0.080	−0.039	0.005	< 0.001	−0.048, − 0.030
HT + GT	− 0.072	− 0.019	0.005	< 0.001	− 0.029, − 0.009
DM + DL	− 0.057	−0.009	0.007	0.178	−0.023, 0.004
HT + GE	−0.068	−0.010	0.007	0.140	−0.024, 0.003
CP + DL	−0.068	−0.018	0.007	0.011	−0.032, − 0.004
BD + DL	−0.069	− 0.016	0.007	0.019	−0.030, − 0.003

Tobit regression models were respectively built and gender, age, education level, marriage state, employment, and all other single diseases were adjusted in the Tobit regression model

^a^ Partial regression coefficient (b) was used to reflect marginal effect HT Hypertension, CP Chronic pain, BD Bone diseases, DM Diabetes mellitus, DL Dyslipidemia, GE Gastroenteritis, GT Gout

## Discussion

### Main findings

Within a sample of Chinese middle-aged and elderly adults, the study results showed that people with chronic conditions were associated with lower HRQoL. Based on this, we found that HRQoL declining to varying degrees was affected by different types and combinations of chronic diseases, and that this decline still persisted following correction for confounding factors. Although the prevalence of hypertension is the highest, its impact on HRQoL is not the greatest. The combination of chronic pain and bone disease has the greatest loss in HRQoL. What’s more, certain chronic diseases often occurred in pairs, especially those pairs involving hypertension or dyslipidemia.

### Comparison with previous studies

The finding, that pain/discomfort was generally the most concerned domain, was line with the previous reports in China [24] and Western developed countries [25]. This suggested that, despite the diversity of populations, the trend remains the same. It could be because the pain/discomfort was the domain which put the greatest limitations on people’s lives, and it was the most important and least bearable condition for them [4]. The differences of HRQoL in age, gender, education level, and marital status in this study were matched with the published literatures [7, 9]. With the age increased, health problems increasing was a common phenomenon, which also affected on HRQoL [26, 27]. Furthermore, the decrease in HRQoL could be caused by marriage problems and it could increase gradually with age for the singles. Because compared with those who are single or divorced, married people have improved mental health due to the social relationship with the spouse [28]. And in our study, female had higher rates of chronic pain and bone disease, the difference by gender was significant. Some studies also showed that females perceived more severe physical damage and poorer health than males [29–31]. HRQoL was lower in females than in males. People with high levels of education can better understand and adhere to the treatment of the disease, and those with lower education levels had more problems related to self-management on a daily basis [32]. HRQoL increased with the improvement of educational level [33]. Thus older single women, those with lower levels of education often reported poorer HRQoL.

In our study, 48.3% adults aged 45 years and above suffered from chronic diseases and 52.5% reported the presence of only one or two chronic condition. Only a few have three or more chronic diseases. The prevalence of multimorbidity increased with age from 12.8% among the middle-aged (45–64 years old) to 33.8% in older adults (over 65 years old), which was lower than that of 20.5 and 45.5% reported in other studies [8, 34]. But in different studies, estimates of the prevalence of multimorbidity varied widely, depending on age of study population, number of involved chronic diseases, measurements, and sampling frame [34, 35]. Although the prevalence of multimorbidity was lower than other studies, the effect of multimorbidity on HRQoL was still significant in the study.

Hypertension was found to be the single most prevalent disease, in line with the actual situation in China [36]. In our study, nearly half of the people who reported a hypertension experienced only one condition, which was different from the study about China of Wang et al. [37]. Perhaps it is because the population selected in this study included the middle-aged, which had a lower rate of multimorbidity compared with older people. Nevertheless hypertension was still the most common in chronic pairs in the study. Then we selected seven prevalent chronic diseases with prevalence rates higher than 4.0% to examine the associations between each other. Almost all of the seven selected prevalent chronic diseases tend to co-occur more often than expected. We detected that six pairs of chronic diseases (hypertension and diabetes mellitus, hypertension and dyslipidemia, dyslipidemia and gout, chronic pain and bone disease, chronic pain and gastroenteritis, and diabetes mellitus and dyslipidemia) were highly correlated. This was consistent with a Japanese study which demonstrated that people with either hypertension or diabetes mellitus had a higher risk of having both conditions [38], and a French study also observed that patients with gout often have comorbid conditions of cardiovascular disease, like dyslipidemia [39]. One study revealed that older adults with diabetes mellitus were significantly more likely to have certain chronic conditions simultaneously, including dyslipidemia [40], which was in agreements with our finding regarding the co-occurrence of diabetes mellitus and dyslipidemia. These findings imply that the primary health care system should provide targeted prevention and treatment program for these co-occur chronic disease.

The associations between chronic diseases and the effect of multimorbidity on the HRQoL made us further explore the impacts of different disease pairs on the HRQoL. Among those chronic diseases with high prevalence, the effects on HRQoL ranged from chronic pain (− 0.036) to hypertension (− 0.006). Previous studies showed that hypertension seemed as a clinically silent disease, impaired the HRQoL, but some other variables had the greater impacts on HRQoL [41]. Chronic pain caused the body or nerves system to continuous or recurrent pain, which had a direct effect on the dimensions of usual activities and pain/discomfort. And there was strong correlation between pain severity and HRQoL [42]. The effects of disease pairs on quality of life were not simply the sum of the effects of two diseases, but had different effects in the combination of different diseases. In the common disease pair, co-occurrence of chronic pain and bone disease (− 0.039) had the greatest impact on HRQoL. But there were some disease pairs leading to a less decline in the quality of life compared with the single disease. That maybe because HRQOL was related to individual’s goals, expectations, and concerns of their life. This implies that the concept of HRQoL is subjective and multidimensional, including positive and negative elements. And with the increasing of diseases, people appeared to become more accustomed to their diseases and to have adapting to them [4]. This allowed them to subjectively reduce the severity of the disease, which accordingly mitigated the impact on HRQoL. Although most literatures agreed that the HRQoL decreases with the increase of the number of chronic diseases [7, 9, 43]. But a single disease which had a great impact on the HRQoL was more serious than a combination of two diseases which had a smaller impact. Therefore, more attention should be paid to diseases and disease combinations that have a greater impact on HRQoL.

### Limitations

There are several limitations in this self-reported study. First, the list of chronic diseases used in this study may have encouraged participants to over-report. And the severity of the chronic illness was not taken into account in this study, which may have an impact on the HRQoL. Second, the community-based survey excluded respondents who were too ill to respond and those in institutions. Third, this study used cross-sectional survey data to analyze the correlations between HRQoL and the associated factors, rather than the causation. Last, we only explored the effects of chronic disease pairs on HRQoL, and the multimorbidity patterns with three or more chronic disease were not taken into account.

## Conclusion

This study provided the associations between chronic diseases, the prevalence of multimorbidity, and the relationships between different multimorbidity patterns with HRQoL, among middle-aged and elderly people in China. The finding of this study had the significant implications for identifying different multimorbidity patients at risk of lower quality of life, and demonstrated the importance of early identification and treatment of diseases. The study also can provide the useful evidence for decision-makers upon optimizing the allocation of health resources, and the health administrative departments should strengthen the management and monitoring of chronic disease, raising awareness of the prevention of chronic diseases. Future research should further explore the relationship of more comprehensive multimorbidity patterns and the quality of life in a prospective cohort study.

## Data Availability

Our data might not be shared directly, because it’s our team work and informed consent should be attained from team members.

## Abbreviations

CI: Confidence interval
HRQoL: Health-related quality of life
SD: Standard deviations
VAS: Visual Analogue Scale
